# The Diagnostic Accuracy of a Novel Scoring System Using Multi-Detector Computed Tomography to Diagnose Lung Cancer

**DOI:** 10.7759/cureus.35848

**Published:** 2023-03-06

**Authors:** Saurav Bhagat, Vishal Gupta, Sujeet Kumar Jain, Sakshi Aaggarwal, Sachin Khanduri, Saumay Batra

**Affiliations:** 1 Department of Radiology, School of Medical Sciences and Research, Sharda University, Greater Noida, IND; 2 Department of Pathology, Faculty of Medicine and Health Sciences, Shree Guru Gobind Singh Tricentenary University, Haryana, IND; 3 Department of Radiology, Era's Lucknow Medical College and Hospital, Lucknow, IND

**Keywords:** cancer, sensitivity, scoring system, lung cancer, multi detector ct

## Abstract

Introduction: Lung cancer is the leading cause amongst the cancer deaths in the world. Detection of malignancy at an early stage and with precision is the utmost objective of radiological evaluation. The final diagnosis of lung cancer is histopathological evaluation of the mass. The authors hereby have tried to convert the multi-detector CT (MDCT) characteristics and patient demographics into quantitative data to formulate a scoring system that can predict lung malignancy as close to histopathology as possible.

Materials and methods: After obtaining ethical clearance, 104 cases of suspected lung cancer by history, clinical and radiographic evaluation were enrolled in the study. These patients were undergoing CT thorax (contrast) on 384 slice siemens somatom force. After undergoing the radiological evaluation biopsy of the mass was done either by CT guided or bronchoscopy guided. Radiological and histopathological findings were correlated. Patients aged >50, lymphadenopathy, tumor volume >50 cc, enhancement >15 HU (Hounsfield unit) after contrast injection were given a score of 15 each. History of smoking, bronchus cut off, spiculated/lobulated margins, mediastinal/pleural involvement, and angiogram sign positive were given a score of 20 each. So, a maximum score of 160 can be achieved by history and MDCT evaluation.

Results: Sensitivity, specificity, positive predictive values (PPV), negative predictive values (NPV), and diagnostic accuracy of MDCT by using conventional parameters against histopathology was 97.5%, 85%, 96.29%, 89.47%, and 95.0%. The sensitivity and specificity calculated through Receiver-Operating-Characteristic (ROC) for predicting malignancy were found to be 98.8% and 90.0% for a cut-off score of >97.5 out of maximum of 160.

Conclusion: MDCT serves as a tool for early diagnosis of lung cancer, and it is the utmost important tool for cases where biopsy or fine needle aspiration cytology (FNAC) is not possible. By creating a quantitative criterion to diagnose lung malignancy, the subjective nature of MDCT diagnosis can be converted into an objective based evaluation.

## Introduction

Lung cancer is the most common kind of cancer-related death in India, accounting for 6.9% of all newly diagnosed cases of cancer and 9.3% of all deaths caused by cancer overall. This statistic includes persons of both sexes. It is the type of cancer that is most prevalent in men and the cancer that is responsible for the majority of deaths in men due to cancer. The number of male and female instances of this sickness that have been documented is greatest in the state of Mizoram [1]. Those who are older than 40 years, and in particular those who are between the ages of 60 and 70, have a significantly increased risk of being diagnosed with lung cancer. People younger than 35 years of age are almost never diagnosed with this illness. There are approximately 1.4 times as many cases of lung cancer in men as in women. This disparity is estimated [2,3]. Multiple detector computed tomography (MDCT), also known as multi-detector CT, often provides more information than chest radiography or normal CT scanning does. MDCT is also known as multi-detector CT. In high-risk groups, screening using low dose computed tomography (CT) showed a relative risk reduction of 20% in lung cancer mortality while having a false positive rate of 96%. For the goal of early illness identification and screening in high-risk groups, the development of novel non-invasive diagnostic techniques or biomarkers is an absolute necessity.

In order to confirm a diagnosis of lung cancer, histopathological information must be obtained from a sample of tissue that was obtained during a biopsy. On the other hand, many positive cases cannot be confirmed due to a variety of factors. Some of these factors include the extremely small size of the tumour, the difficulty in reaching the location of the tumour, severely ill patients, and the refusal of patients to undergo invasive procedures such as bronchoscopy, biopsy, or surgery. Consequently, many positive cases go unconfirmed [4]. It may not always be possible to acquire a biopsy, particularly in situations in which a tumour is extremely small or is situated in a location that is difficult to reach. As a result, an alternative method for accurately identifying lung cancer was necessary, in particular for conditions in which histological examinations are impossible to be performed. The existence of such a malignant tumour can be identified with a chest CT that makes use of a technique known as high resolution computed tomography [5,6]. This study aimed to design a scoring system for MDCT that would be capable of predicting lung cancer with an accuracy comparable to that of a histopathological examination. This objective was the driving force behind the study.

## Materials and methods

We carried out a cross sectional observational study in the North Indian population with an institutional ethical clearance number ELMC/EC/RCELL/2014/219. In this study, we enrolled a total of 104 patients who were seeking treatment at the radiodiagnosis department and had a medical history and clinical presentation that were suggestive of the presence of a lung mass. Every single patient presented with symptoms that strongly suggested the existence of a lung tumour. Even though the results of four biopsies came back inconclusive, the research was carried out with a total of one hundred individuals since it was necessary to preserve statistical significance.

**Figure 1:**
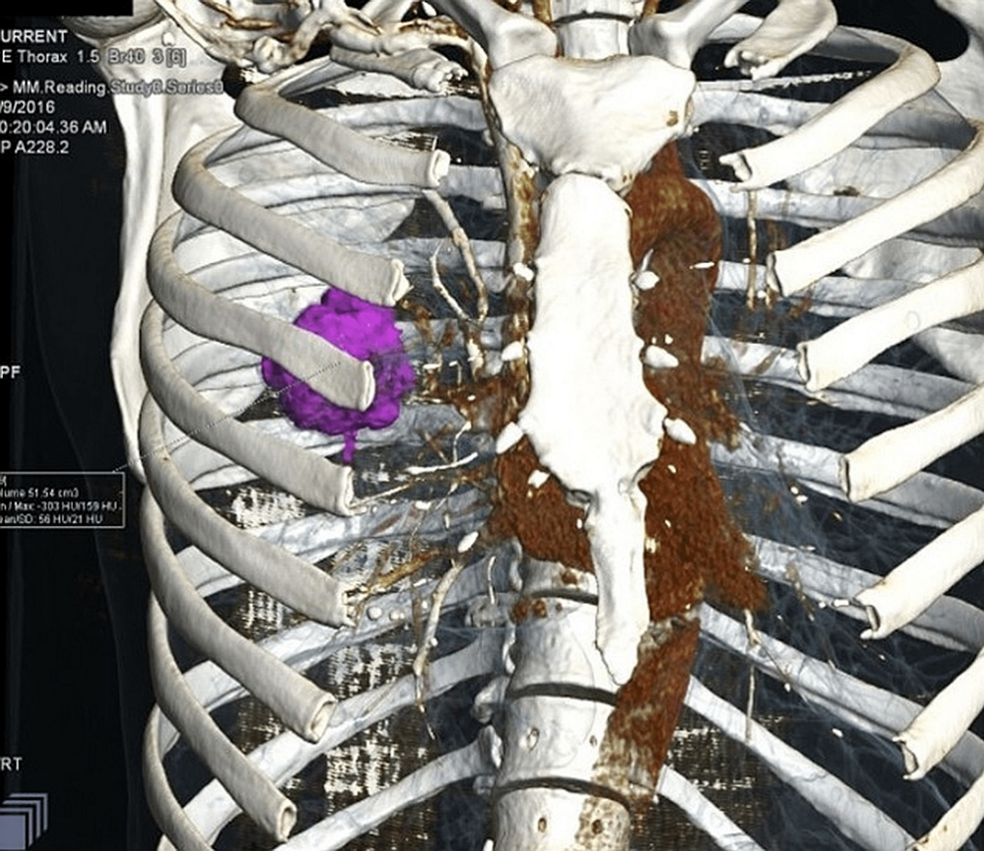
Tumor volume as calculated by VRT image VRT: Volume Rendering Technique

This inquiry was undertaken for two years. During the course of the study, an assessment of the results produced by the multi-detector computed tomography was used in part of the work that was done. The following observations were obtained and enumerated: the volume of the tumour, spiculation, pleural extension, angiography sign, bone involvement, metastasis, and the expansion of the lymph node. Two important groups of anomalies discovered in CT imaging were investigated in this study (Figure 1).

The majority of the patients fell into one of these categories. The primary abnormalities, which included 10 things, and the supplemental abnormalities, which included eight items, may be broken down into these categories. In this particular investigation, both groups of anomalies were considered to be subjected to evaluation. In the research carried out by Abdullah and colleagues [4], these aberrations were further categorized as in Table 1.

**Table 1:** Scoring to predict lung cancer

	Characteristic Score		Score
Age	<50	0	
	≥50	1	15
Smoking	No	0	
	Yes	1	20
Lymphadenopathy	Absent	0	
	Present	1	15
Tumor Volume	<100 cm^3^	0	
	>100 cm^3^	1	15
Enhancement	<20	0	
	>20	1	15
Bronchus cut off	Absent	0	
	Present	1	20
Spiculation	Absent	0	
	Present	1	20
Mediastenal /Pleural Involvement	Absent	0	
	Present	1	20
Angiogram	Absent	0	
	Present	1	20
Total Score			160

As additional diagnostic criteria recorded during the course of the inquiry, the attending pathologist documented the histological examination of sputum, bronchoscopy, and lymph node biopsies. These were all performed as part of the investigation. Imaging with MDCT was carried out in a manner that was consistent with the procedures outlined by Ng et al. [7]. In order to create a grading system, the outcomes of the MDCT as well as the histopathology results were added to the demographic information that was already there. Smoking, lymphadenopathy, tumour volume (>100 cubic centimeter), enhancement (>20), bronchus cut off (+), spiculation +, mediastinal/pleural involvement, and angiogram+ each have a value of respectively 15, 20, 15, 20, 15, 20, 15, 20, 20, 20, and 20, for a total score of 160. This score is based on patients who are 50 years old or older. In each case, it was determined that the disease had already spread to other bones and that it was a malignant kind.

The MDCT examination and the findings of the histological investigation were compared using bivariate tables, and the analysis of these tables was performed using a diagnostic tool known as a 2x2 table. We used version 20.0 of the Statistical Package for the Social Sciences (SPSS) software (IBM Corp., Armonk, NY) to analyze the data in order to calculate sensitivity, specificity, positive predictive value, and negative predictive value. Calculations using many variables utilizing SPSS version 20.0 were carried out in order to establish variables with particular values. These calculations were carried out in order to determine the scores required to compare the MDCT imaging of main abnormalities and additional lesions to the findings of the histopathological examination. Additionally, these calculations were carried out in order to take into account a demographic feature known as age. The completion of the score value was followed by the establishment of a cut-off point in order to discriminate between benign and malignant diseases. The final score was determined by adding the results of all of the computations performed on the various factors.

## Results

A summary of the demographic and clinical characteristics of the patients who were studied at the beginning of the research may be seen in Table 2 and Table 3, respectively.

**Table 2:** Demographic data

Variable	Mean ±SD	95% Confidence intervals
Age(year)	54.45±10.48	52.37 – 56.53
Gender (Male :Female)	17:8	-
Zone (Rural :Urban)	39:11	-

**Table 3:** Smoking cases

Smoking	Present	Absent
	70 (70%)	30 (30%)

The results of the histological analysis revealed that adenocarcinoma was the most common type of cancer, making up 38 (38%) of the cases altogether. The number of benign instances accounted for twenty (20%) of the diagnoses, whereas the number of cases of small cell carcinoma accounted for sixteen (16%) of the diagnoses. In this specific study, the findings of the MDCT on 19 patients were determined to be benign, and the findings of the pathology on 17 patients (90.9%) concurred with the MDCT findings as benign lesions (Table 4).

**Table 4:** Clinical features

Variable	Present	Absent
Cough	91 (91%)	9 (9%)
Sputum	78 (78%)	22 (22%)
Breathlessness	30 (30%)	70 (70%)
Fever	65 (65%)	35 (35%)
Hemoptysis	67 (67%)	33 (33%)
Chest pain	26 (26%)	74 (74%)
Weight loss	48 (48%)	52 (52%)

Only two of the individuals showed any evidence of having cancerous cells. On the other hand, cancer was discovered in 81 of the remaining individuals who had MDCT. The findings of the MDCT and the histology were in agreement in 78 out of 80 patients (an agreement rate of 95.5%), and just three patients had benign findings. When the results of the MDCT and the histopathology were compared side by side, it was found that the MDCT method had an accuracy of 95%, a sensitivity of 97.5%, a specificity of 85%, a positive predictive value of 96.29%, and a negative predictive value of 89.47% (Tables 5, 6).

**Table 5:** Histopathology diagnosis

Variable	Number
Adenocarcinoma	38
Benign	20
Large cell carcinoma	6
Sarcomatoid	2
Small cell carcinoma	16
Squamous cell	18

**Table 6:** Comparison of histopathology and MDCT MDCT: Multi-detector computer tomography

Computed tomography	Histopathology Examination		
	Malignant	Benign	Total
Malignant	78	3	81
Benign	2	17	19
Total	80	20	100

This was determined by comparing the two sets of results side by side. The scores produced from the examination of the scoring system were used as the basis for an application of the Receiver-Operating-Characteristic (ROC) analysis. This study, which uses the cutoff value (sensitivity and specificity) as the criterion for finding the crosspoint, was used to determine the crosspoint. This analysis is known as a cutoff value analysis. The researchers came to the conclusion that cancer could be predicted with a sensitivity of 98.8 percent and a specificity of 90 percent when they reached the cross point of 97.5. The cutoff criterion was determined by taking into account the values of sensitivity and specificity that performed the best in terms of reliably predicting instances of lung cancer. This was done in order to construct the criterion.

## Discussion

The chest radiograph is currently, and will continue to be in the foreseeable future, the most important imaging method for lung parenchyma. It is unrivalled in terms of the amount of information it gives regarding the amount of radiation exposure, as well as the fact that it is inexpensive, readily available, and convenient to carry out. However, there are a few limitations that come along with the chest radiograph. About 10-15 percent of symptomatic individuals verified to have infiltrative lung disease have normal findings; about 30-60 percent of patients with bronchiectasis have normal levels; and about 10-15 percent of patients who have emphysema have normal levels. In a number of examinations, it was discovered that the chest radiograph "had an overall sensitivity of 80 percent and a specificity of 82 percent for the diagnosis of diffuse lung disease. Chest radiography was the only method that resulted in a confident diagnosis being achieved in 23 percent of the cases, and those confident diagnoses were only verified to be accurate in 77 percent of the cases.

Despite this, the investigations on MDCT screening that have been carried out up to this date have corroborated some aspects of the chest radiography study. Despite the fact that MDCT identifies nearly 10 times the number of early lung cancers compared with control groups, this screening technology did not lower the prevalence of advanced lung malignancies [8-11]. In fact, several studies found a small number of additional advanced cancers in comparison with the values that were expected [12].

Because none of the nonsurgical treatment options was successful in collecting enough tissue for pathological examinations, the histological subtype of around 10-20% of lung cancer cases cannot be established with certainty. When the diagnosis of lung cancer has not yet been confirmed, it is impossible to provide appropriate treatment for the condition until surgery has been performed. For this reason, it is essential to have a noninvasive method that is just as accurate as pathological examinations for circumstances such as these. To the best of our knowledge, this study is the first to design an MDCT scoring system that has the potential to predict lung cancer with an accuracy that is close to that of histological examination.

When the diagnosis of lung cancer has not yet been established, it is difficult to give suitable therapy for the condition until surgery has been conducted. For this reason, it is essential to have a noninvasive method that is equally as accurate as pathological examinations for scenarios such as these. To the best of our knowledge, our study is the first to establish an MDCT scoring system that has the potential to predict lung cancer with an accuracy that is near to that of histological inspection by Arora et al. [13], Krishnamurthy et al. [14] and Yadav et al. [15]. The most common symptom was a cough, followed by the production of sputum in 78% of cases, hemoptysis in 67%, and fever in 65% of cases [15]. The most common symptom was a cough, which was experienced by 23 out of 25 patients (83.3%), followed by dyspnea, which was experienced by 24 out of 25 patients (80%), weight loss, which was experienced by 20 out of 25 patients (66.6%), chest pain, which was experienced by 16 out of 25 patients (53.3%), hemoptysis, which was experienced by 9 out of 25 patients (30%), hoarseness of voice, which was experienced by 6 out of 25 patients (20%), as shown by Rawat et al. [16].

Quantitative perfusion CT techniques have only been able to be applied to a single tumour level up until this point in time. However, because of advancements in MDCT scanner technology, it is now feasible to take a series of continuous axial photographs at the same level as the tumour. The configuration of the rows will have an effect on the photographs that are taken. For example, with a 16-MDCT scanner, it is feasible to capture four continuous photos with a length of 6 millimetres along the axial direction. This yields a coverage of 24 millimetres along the z-axis. On the other hand, it is a well-established fact that the vasculature of tumours demonstrates both spatial and temporal variability. This indicates that blood flow may alter at different times and in different locations inside a single tumour, depending on the microenvironment of the tumour. These changes can occur at different points in time [17,18]. This was demonstrated in the current study after the results were analysed. It showed that the MDCT method had an accuracy of 95% when compared to the results of the histopathology. It also had a sensitivity of 97.5 percent, a specificity of 85 percent, a positive predictive value of 96.29%, and a negative predictive value of 89.47%. During a diagnostic procedure that does not include surgery, the major factor that establishes whether or not a patient has lung cancer is whether or not they have large cells packed with lipids [19-21]. On unenhanced CT scans, adenomas can be recognised by having a Hounsfield unit (HU) level lower than 10. The research that was conducted by Mathieson and colleagues revealed that this particular criterion has a sensitivity of 71% while also having a specificity of 98%. When additional characteristics, such as size, are taken into account, the specificity comes close to reaching 100% (10 on CT scans) [22]. In the study that was carried out by Li et al. using 20-second imaging and 2-dimensional region of interest analysis, the researchers discovered that a 30-HU enhancement or greater had a sensitivity for malignancy of 99%, with a specificity of 54%, a positive predictive value of 71%, and a negative predictive value of 97% [19]. Additionally, the researchers found that this level of enhancement had a positive predictive value of 71% and a negative predictive value of 97%. In addition, the researchers discovered that a 30-HU boost or larger had a positive predictive value of 71%, while a negative predictive value of 97% was shown to have a far higher accuracy. The implementation of image-processing methods such as volumetric enhancement analysis and semi quantitative enhancement maps has the potential to yield benefits for the evaluation of contrast-enhanced data for nodule perfusion. These benefits have the potential to be realised [19,20].

In recent Indian study done by Yadav et al. [15], the total number of genuine positive cases was recorded at 28, while the number of false positive cases was two. There was not a single instance that fit either the criteria for a true negative or a false negative (Note: As a consequence of this, the CT sensitivity was computed to be 100%, which is a significantly higher value than that which was discovered in the present investigation due to the small sample size). On the other hand, the accuracy of the CT was approximately estimated to be 93.3 percent. As a result, the value of making a correct prediction was 93.3 percent. The fact that the positive predictive value of MDCT in evaluating bronchogenic cancer was 93.3% demonstrates that it is, in fact, a useful tool in the process of evaluating bronchogenic carcinoma. In the research that Baron and his colleagues conducted, they discovered that the prediction of CT sensitivity was 93% accurate [21].

In a study by Mathieson et al. [22], the accuracy of computed tomography (CT) was compared to that of simple chest radiography in patients diagnosed with chronic diffuse infiltrative lung disease. When compared to the accuracy of chest X-rays, the authors of the study discovered that the diagnostic accuracy of CT for lung cancer was 93%. This level of accuracy is statistically significant when considering how accurate CT is. The highest correct interpretations of the CT scan were seen in instances of silicosis (93%), typical interstitial pneumonia (89%), lymphangiticcarcinomatosis (85%), and sarcoidosis (77%). In addition, Grenier and his colleagues discovered that CT is beneficial in identifying the accurate diagnosis in individuals who have CDILD [23].

In a study by Kim et al. [24], low-dose chest CT and calcium-scoring CT with prospective ECG gating utilising 40-MDCT were performed on the subjects, all of whom were males and had a mean age of 52 +/- 7 years. One hundred twenty-eight individuals have been included in this study in a sequential fashion. Reconstructed were the results of a low-dose chest CT that had a field of view of 25 centimetres and three slice thicknesses of 1, 2.5, and 5 millimetres each. The researchers discovered that using a slice thickness of 1 millimetre at 130 helium and 2.5 millimetres at 90 helium was the most effective technique to measure sensitivity in low-dose chest CT scans. This was the case regardless of the helium pressure. Utilizing a slice thickness of 5 millimetres at an angle of 130 degrees proved to be the most effective way of assessing specificity. A temperature of 130 degrees Celsius and a slice thickness of 2.5 millimetres provided the best circumstances for achieving an accuracy of 90 percent.

In the Takanashi study, the tumours were smaller than 2 cm, which indicates that the cell volume was only 8 cm^3^, and the size of the tumours was less than 2 cm [25]. When it comes to identifying adenomas, the magnetic resonance (MR) method reportedly has a very high degree of accuracy, with reported sensitivities ranging from 81% to 100% and specificities ranging from 94% to 100%. This information comes from Mathieson et al., who state that the method has a very high degree of accuracy [22]. During the course of this study, we developed a scoring method, which is essential for converting the anomalies into numbers that can be computed to show whether or not they point to the presence of cancer. In other words, we were able to determine whether or not the anomalies indicated the presence of cancer. This research was successful in developing a system that translates the primary abnormalities and extra lesions found on MDCT, together with the demographic element of age, into scores that can be used to differentiate between conditions that are cancerous and those that are not cancerous. Patients diagnosed with thymoma were included in the current study because it was difficult to tell from a chest X-ray where the masses in the mediastinum and hilar region originated.

## Conclusions

Since it has a higher spatial resolution than other imaging modalities, multi-detector computed tomography has emerged as the imaging modality of choice for the evaluation of lung cancer. This is because of its ability to detect smaller tumors. In light of this fact, the computed tomography (CT) scan plays a very significant part in the characterization of the suspicious lesion's size, shape, and extent, in addition to the tissue composition of the lesion itself. Additionally, it has been demonstrated, through the utilization of the MDCT scoring system, that the scores are equally as accurate as the results of the histology.
